# Whole-Embryo 3D Quantification Reveals Conserved Topological Design and Scaling of Germ Layers in Xenopus

**DOI:** 10.64898/2026.06.16.732511

**Published:** 2026-07-03

**Authors:** Hugo M. Santos, Maria Diakova, Max Brambach, Cora Anderson, Kseniya Petrova, Cecilia A. De Araujo, Iva Simeonova, Geneviève Almouzni, Leonid Peshkin, Jose G. Abreu

**Affiliations:** 1: Instituto de Ciências Biomédicas, Universidade Federal do Rio de Janeiro, Cidade Universitária, Rio de Janeiro, RJ 21941-902, Brazil.; 2: Systems Biology Department - Harvard Medical School, Boston, MA, USA.; 3: Institut Curie 75005, Paris, France; CNRS (French National Center for Scientific Research), Nuclear Dynamics unit UMR3664, Paris, France; Sorbonne Université 75005, Paris, France; PSL Research University, Paris, France.

**Keywords:** Germ-layer Topology, Xenopus Cell Atlas, Vertebrate Embryogenesis, *In-toto* 3D Imaging, Developmental Invariants, Deep-Learning Nuclei Segmentation

## Abstract

How embryos of different sizes generate reproducible body plans remains a central question in developmental biology. Do larger embryos contain more cells, or preserve conserved organizational principles that ensure robust tissue patterning independent of scale? Here, we address this question through whole-embryo quantitative mapping of cell number, tissue allocation, and spatial organization during early development in Xenopus. Using optimized in-toto 3D imaging, tissue clearing, and deep-learning for nuclei segmentation, we quantified cell numbers and reconstructed the spatial distribution of cells in early embryonic stages. Although X. laevis embryos exhibited substantially larger embryo volumes and higher total cell numbers than X. tropicalis, the proportional allocation of cells among ectoderm, mesoderm, and endoderm remained highly conserved between species. In addition, quantitative analysis of local cellular neighborhoods revealed striking conservation of spatial order, packing geometry, and large-scale tissue architecture despite major differences in embryo size and cellular density. Together, these findings demonstrate that early vertebrate embryos follow shared quantitative design principles in which embryonic scaling occurs without disruption of the underlying cellular blueprint of the body plan. Our study establishes a quantitative framework for comparing embryonic architecture across species and provides evidence that developmental organization is governed by conserved scale-invariant topological principles.

## INTRODUCTION

One of the most fundamental questions in developmental biology is how embryos of vastly different sizes reliably produce identical body plans. This phenomenon—developmental scaling—implies the existence of robust mechanisms that coordinate cell number, tissue size, and spatial organization across species and across individuals within a species (Čapek & Müller, 2019; Wolpert, 1969). During the transition from gastrulation through neurulation to the tailbud stage, the three primary germ layers—ectoderm, mesoderm, and endoderm—are progressively specified, allocated, and patterned to architect the vertebrate body axis. Whether these processes scale linearly with cell number or rely on conserved topological “design rules” that are independent of absolute embryo size remains poorly understood.

Classic experimental studies demonstrated that embryonic form can remain remarkably robust despite major alterations in cell size and cell number. In pioneering work, Fankhauser showed that polyploid amphibian embryos develop with fewer but substantially larger cells while maintaining nearly normal tissue and organ dimensions (Fankhauser, 1945; Fankhauser, 1972). Fankhauser’s experiments on newt embryos showed that making cells larger by increasing ploidy did not lead to a corresponding increase in the overall size of the animal. Instead, tissues and organs such as the pronephric duct retained its normal dimensions, even though the number of cells forming the duct dropped dramatically—in pentaploid embryos as few as one to three cells had to enclose a lumen that normally required three to eight cells. More recent studies in vertebrate and invertebrate systems have further shown that morphogen gradients, tissue proportions, and developmental dynamics can adapt to changes in embryo size through scale-invariant regulatory and mechanical mechanisms (Ben-Zvi et al., 2011; Almuedo-Castillo et al., 2018; Heisenberg & Bellaïche, 2013). Together, these findings suggest that embryonic development may rely on conserved organizational principles that preserve tissue architecture independently of absolute embryo size or cell number. However, direct whole-embryo quantitative tests of this idea at cellular resolution remain limited.

The genus *Xenopus* provides an exceptional comparative framework for addressing this question. *Xenopus laevis*, a pseudotetraploid with large, yolk-rich eggs (~1.2 mm diameter), and *Xenopus tropicalis*, a diploid with smaller eggs (~0.7 mm), exhibit substantial differences in egg size, genome content, cell volume, and total embryonic volume (Levy & Heald, 2010; Gibeaux et al., 2018). Despite these differences, both species follow nearly identical developmental trajectories to produce functionally equivalent tadpoles. This natural experiment in embryonic scaling—two closely related species with different absolute sizes but conserved developmental programs—makes *Xenopus* an ideal system to ask: do larger embryos simply have more cells, or do they follow common design rules?

The inherent opacity of Xenopus embryos has historically limited quantitative approaches to *this* question. Unlike the transparent embryos of zebrafish or the accessible embryos of mice, *Xenopus* embryos are densely packed with yolk platelets and pigmented granules that scatter and absorb light, precluding direct deep-tissue 3D imaging (Miller et al., 2023). Early quantitative studies by Cooke (1979a, 1979b) established that cell number in *Xenopus laevis* is tightly regulated during gastrulation and neurulation, with sequential recruitment of cells into the mesoderm and spatially patterned mitotic activity. However, these studies relied on manual counting and serial sectioning, limiting both throughput and spatial resolution. More recently, advances in tissue clearing—particularly the iDISCO family of protocols—combined with automated deep-learning segmentation tools such as StarDist3D have made it possible to perform whole-embryo, single-nucleus quantification in 3D (Renier et al., 2014; Schmidt et al., 2018).

In this study, we developed an optimized pipeline for *Xenopus* whole-embryo 3D imaging, combining hydrogen peroxide bleaching for pigment removal, nuclear staining with multiple fluorescent probes, and iDISCO clearing in ethyl cinnamate. We then applied StarDist3D deep-learning segmentation to quantify the absolute number of cells and their spatial distribution across the three germ layers at stages 11.5 (gastrula), 15 (neurula), and 23 (early tailbud) in both *X. laevis* and *X. tropicalis*. Our results address three core questions: (1) Is there a proportionality in the number of cells within each embryonic tissue across different species? (2) Are cellular metrics—such as spatial order, packing, geometry, and directionality—conserved across different developmental stages? (3) Do the different species share quantitative self-organization principles?

Our data reveal that while *X. laevis* is substantially larger in both total volume and cell count, the spatial organization and topological architecture of the embryo are remarkably conserved between species. These findings suggest that early vertebrate embryos follow shared quantitative design principles—a “cellular blueprint”—in which cell-number scaling occurs without disrupting the fundamental organization of the developing body plan.

## RESULTS

### 1. Development of an Optimized Pipeline for Whole-Embryo 3D Imaging in *Xenopus*

**Figure 1.**
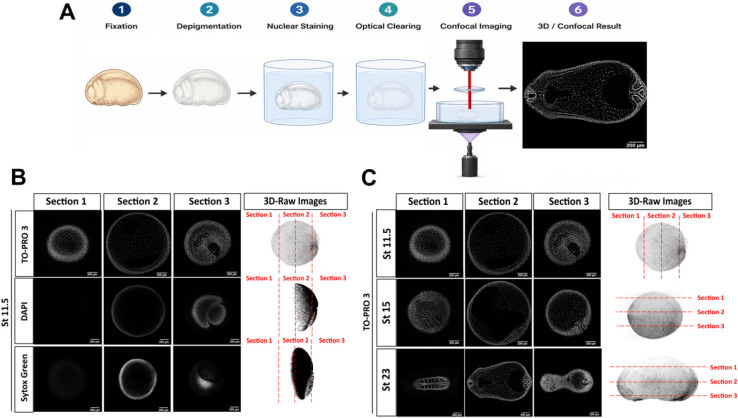
High-resolution *in toto* 3D imaging of *Xenopus* embryos enabled by far-red nuclear staining. **(A)** Schematic workflow of the *in toto* sample preparation protocol: MEMFA fixation, H_2_O_2_ depigmentation, TO-PRO-3 nuclear staining coupled with ethyl cinnamate (ECi) optical clearing, and spinning disk confocal sectioning. **(B)** Comparative assessment of nuclear fluorophores in gastrulating embryos (St. 11.5). TO-PRO-3 exhibits superior deep-tissue penetration compared to DAPI and Sytox Green. Its far-red excitation (~640 nm) successfully overcomes physical light scattering in dense embryonic tissues, enabling complete and uniform 3D volumetric reconstructions. **(C)** Validation of the optimized protocol across key developmental stages: gastrulation (St. 11.5), neurulation (St. 15), and larval stage (St. 23). Consecutive optical sections and 3D projections confirm consistent cellular-level resolution and structural preservation throughout the entire embryo volume. Scale bars = 200 μm.

A major challenge in quantifying *Xenopus* embryo architecture is the inherent opacity of the tissue. The high yolk content, dense pigmentation, and autofluorescence of cells, particularly at early stages, severely limit light penetration and confocal imaging depth. To overcome these limitations, we used currently available methods (Mazière et al., 1996; Masselink and Tanaka, 2023) and developed a multi-step preparation protocol optimized for *Xenopus* embryos at stages 11.5, 15, and 23 (Figure 1A).

Embryos were first bleached with hydrogen peroxide to remove pigmentation, then subjected to whole-mount nuclei staining. We systematically evaluated three fluorescent nucleic acid probes: SYTOX Green, DAPI, and TO-PRO3, which have emission peaks at 523 nm (Green), 461 nm (Blue/Cyan), and 661 nm (Far-Red), respectively. Following staining, embryos were processed using the iDISCO protocol (adapted from idisco.info, Renier et al., 2014) with ethyl cinnamate as the final clearing medium, achieving near-complete optical transparency while preserving nuclear morphology. The cleared embryos were then subjected to optical sectioning by spinning disk confocal microscopy. This approach preserves the embryo's overall morphology while enabling nuclear signal detection.

We next compared the performance of different nuclear fluorophores in gastrulating embryos at stage 11.5 (Figure 1B). TO-PRO-3 produced a strong and well-defined nuclear signal across multiple optical sections, including superficial, intermediate, and deeper regions of the embryo. In contrast, DAPI and Sytox Green showed more limited signal detection, particularly in deeper regions of the embryo volume. The 3D raw reconstructions shown in Figure 1B further demonstrate that TO-PRO-3 resulted in more homogeneous signal penetration throughout the embryo. In contrast, DAPI and Sytox Green displayed weaker or more restricted deep-tissue labeling. These results indicate that TO-PRO-3 is better suited for whole-embryo nuclear imaging in *Xenopus*, consistent with the improved tissue penetration expected from its far-red excitation wavelength.

Finally, we applied the TO-PRO-3-based imaging strategy to different developmental stages, including gastrulation, neurulation, and larval development (Figure 1C). Representative optical sections from stage 11.5, stage 15, and stage 23 embryos showed nuclear labeling throughout the embryo volume. The corresponding 3D raw images demonstrated that the method can be applied to embryos with distinct morphologies, from the rounded gastrula and neurula stages to the elongated larval body at stage 23. At each stage, a nuclear signal was detected with cellular-level resolution across the embryo, supporting the use of this approach for whole-body 3D reconstruction and quantitative analysis of *Xenopus* embryogenesis.

Together, these results identify TO-PRO-3 as the most effective nuclear dye among those tested. At the same time, the developmental series (Figure 1C) shows that this strategy is compatible with multiple embryonic stages and preserves cellular-level nuclear resolution throughout the embryo volume.

### 2. Validation of Automated Deep-Learning–Based Segmentation with StarDist3D

**Figure 2.**
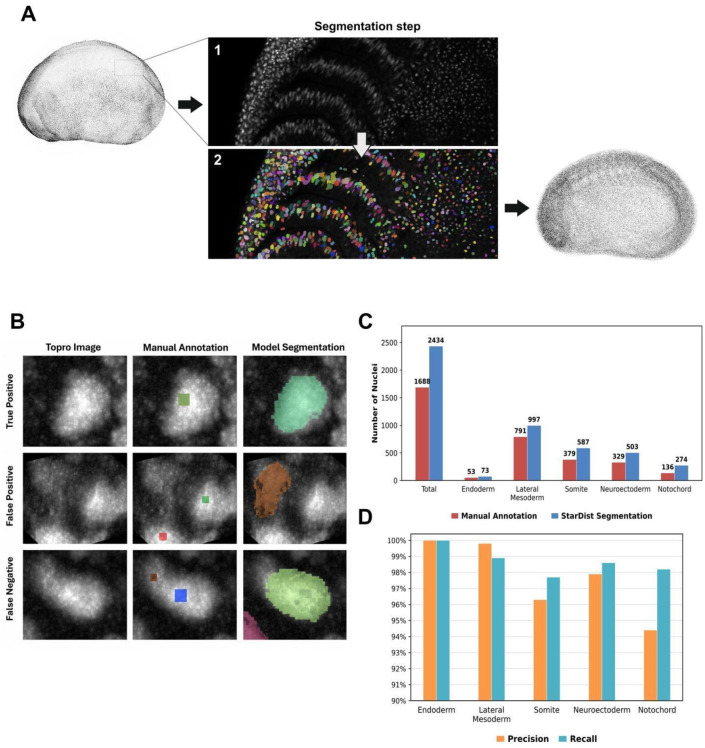
Quantitative validation of StarDist3D nuclear segmentation across diverse embryonic tissues elucidates high accuracy. **(A)** 3D segmentation pipeline using StarDist3D to convert raw optical sections (1) into nuclear masks (2) and a complete 3D spatial model. **(B)** Visual validation comparing manual annotations and model predictions across True Positive, False Positive, and False Negative instances. **(C)** Absolute cell counts. Automated segmentation (blue) systematically detects more nuclei than manual counting (red), overcoming human underestimation in dense tissues. (D) Quantitative metrics. Precision and Recall remain robust (>93%) across all analyzed embryonic structures.

To quantify absolute cell numbers from 3D volumetric images, we implemented and validated StarDist3D, a deep-learning framework designed for instance segmentation of star-convex objects such as cell nuclei (Schmidt et al., 2018; Weigert et al., 2020). Rather than training a model from scratch, nuclear segmentation was performed using a robust, pretrained StarDist3D model originally trained on annotated 3D fluorescence microscopy datasets of cell nuclei. We used this pretrained model to segment our data, validating its performance against manually annotated *Xenopus* nuclear images to ensure it accurately captured the specific size, shape, and packing characteristics of embryonic nuclei at each stage (Figure 2A).

Validation against manual counts in representative regions demonstrated that the pretrained StarDist3D model achieved exceptional segmentation accuracy, specifically overcoming the limitations of manual annotation. In dense embryonic tissues, human annotators systematically underestimate cell numbers due to 3D visual overlap—a well-recognized limitation (Cooke, 1979a). Our automated pipeline provided a robust, highly sensitive alternative. For instance, comparative quantitative analysis revealed that StarDist3D systematically detected a higher absolute number of nuclei (2,434 total nuclei) compared to manual counting (1,688 total nuclei) within the same volumetric regions (Figure 2C). This enhanced detection capability was consistently observed across diverse embryonic structures, including the endoderm, lateral mesoderm, somites, neuroectoderm, and notochord.

Importantly, this increased detection rate did not compromise accuracy. Quantitative evaluation of the segmentation efficiency confirmed that both precision and recall remained exceptionally robust (exceeding 93%) across all analyzed tissues, achieving near-perfect performance in structures such as the endoderm (Figure 2D). By effectively capturing true positives while minimizing false negatives in complex, overlapping tissue environments (Figure 2B), the model confirmed highly consistent performance. The validated pipeline was subsequently applied to the full embryo datasets for comprehensive cell number quantification.

### 3. Absolute Cell Number and Volumetric Scaling from Gastrula to Early Tailbud

**Figure 3.**
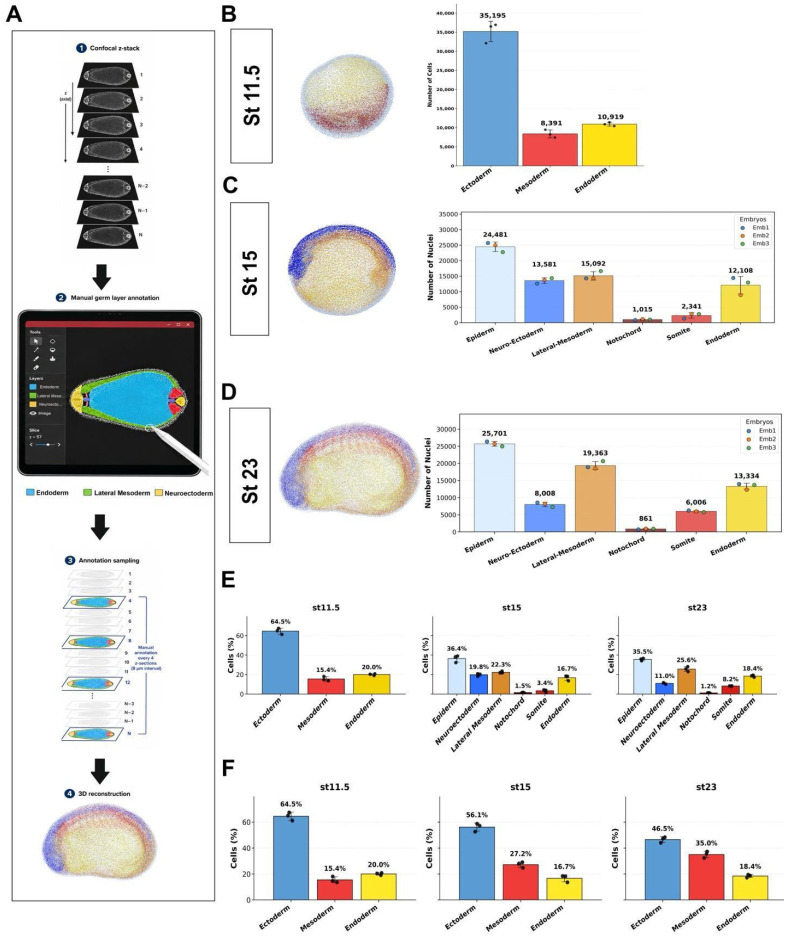
Whole-embryo quantification tracks spatiotemporal cellular dynamics from gastrulation to larval stages in *Xenopus laevis*. **(A)** 3D spatial classification pipeline: confocal z-stacks (1) are manually annotated in Napari (2) every 8 μm (3) and intersected with nuclear coordinates to map germ layer fates (4). (B-D) Absolute cell counts within specific tissues during **(B)** gastrulation (St. 11.5), **(C)** neurulation (St. 15), and **(D)** larval stage (St. 23) (n = 3, mean ± SD). **(E)** Relative cellular composition (%) across detailed sub-tissue layers. **(F)** Normalized distribution consolidated into the three primary germ layers (Ectoderm, Mesoderm, Endoderm) over developmental time.

To track spatiotemporal cellular dynamics from gastrulation to the larval stage in *Xenopus laevis*, we implemented a comprehensive 3D spatial classification pipeline (Figure 3A). Confocal z-stacks were manually annotated in Napari at 8 μm intervals and subsequently intersected with extracted 3D nuclear coordinates to map individual cells to their respective germ-layer fates. Using this approach, we quantified absolute cell counts across distinct tissue lineages during three key developmental milestones: stage 11.5 (gastrulation), stage 15 (neurulation), and stage 23 (larva) (Figure 3B–D; n = 3, mean ± SD).

Our data reveal robust, lineage-specific proliferation and dynamic tissue allocation driving these morphological transitions. During gastrulation (st 11.5), the embryo is predominantly composed of ectodermal cells, which account for 64.5% of the total quantified cells, followed by the endoderm (20.0%) and mesoderm (15.4%) (Figure 3B, E). As development progresses through neurulation (st 15) and into the larval stage (st 23), our pipeline captures the specification of these primary layers into detailed sub-tissues. This includes the subdivision of the ectoderm into epidermis and neuro-ectoderm, as well as the regionalization of the mesoderm into lateral-mesoderm, notochord, and somites (Figure 3C–E).

Consolidating these detailed lineages back into the three primary germ layers highlights significant proportional shifts over developmental time. Most notably, from stage 11.5 to stage 23, the overall normalized proportion of ectoderm-derived cells decreases from 64.5% to 46.5%, whereas the mesodermal fraction undergoes a substantial expansion from 15.4% to 35.0% (Figure 3F). Together, these absolute and relative quantifications demonstrate a clear spatiotemporal progression of cellular allocation as the embryo develops.

### 4. Proportional Tissue Allocation Across Germ Layers

**Figure 4.**
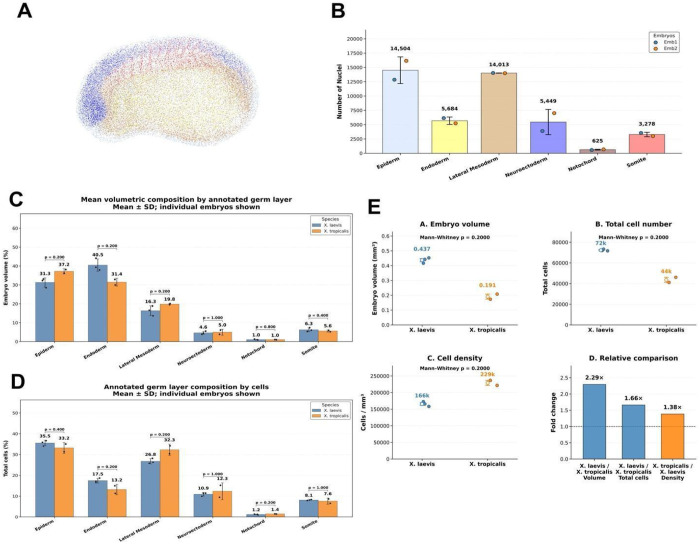
Interspecies 3D morphometric analysis reveals divergent scaling but conservative tissue organization between *Xenopus laevis* and *X. tropicalis*. (A) Color-coded 3D spatial reconstruction mapping *X. tropicalis* (St. 23) nuclei to tissue layers. (B) Absolute cell counts across *X. tropicalis* tissues (n = 2, mean ± SD). Interspecies comparison of (C) relative tissue volume (%) and (D) cellular composition (%). (E) Global macrostructural metrics. *X. laevis* displays larger total volume (2.29x) and cell count (1.66x), whereas *X. tropicalis* exhibits greater cellular packing density (1.38x). Mann-Whitney U test p-values are indicated.

To determine whether the proportionality of cell allocation among the three germ layers is conserved between species despite differences in absolute size, we performed a 3D spatial classification of segmented nuclei into annotated tissue layers at the larval stage (stage 23) in *X. tropicalis* (Figure 4A).

We next assessed whether these divergent physical scales and distinct cellular packing densities influence structural organization. The spatially classified nuclei were mapped to specific compartments, including the epiderm, endoderm, lateral mesoderm, neuroectoderm, notochord, and somites (Figure 4B). Whether measured by mean volumetric composition (Figure 4C) or by total cellular composition (Figure 4D), the relative proportions of ectodermal, mesodermal, and endodermal tissues showed no statistically significant differences (Mann-Whitney p ≥ 0.300) between *X. laevis* and *X. tropicalis*.

Using our validated volumetric segmentation pipeline, we first quantified the embryos' global macrostructural metrics at this stage. *X. laevis* embryos exhibited a significantly larger global scale, with a 2.29-fold greater total embryo volume compared to *X. tropicalis* (0.437 mm^3^ versus 0.191 mm^3^, respectively) (Figure 4E). This volumetric expansion is accompanied by a 1.66-fold higher total cell count in *X. laevis* (72,000 versus 44,000 cells).

Importantly, these scaling factors are not linear; the 1.66-fold increase in cell number does not match the 2.29-fold increase in total volume. This discrepancy indicates that *X. laevis* cells are intrinsically larger at this equivalent developmental stage, consistent with the known relationship between egg size, cell size, and ploidy in *Xenopus*. Consequently, *X. tropicalis* embryos display a 1.38-fold higher cellular packing density (229,000 cells/mm^3^ compared to 166,000 cells/mm^3^ in *X. laevis*), reflecting a much more compact physical arrangement of cells within the smaller embryonic volume.

This robust proportionality in tissue allocation at stage 23 indicates that the physical and biological mechanisms governing germ layer specification and tissue formation operate in a strictly scale-invariant manner. The developmental blueprint is maintained regardless of absolute cell number or packing density, an observation highly consistent with the proportional, scale-invariant growth observed in other model organisms following cellular perturbation.

### 5. Conservation of Spatial Cellular Architecture: Packing, Order, and Geometry

**Figure 5.**
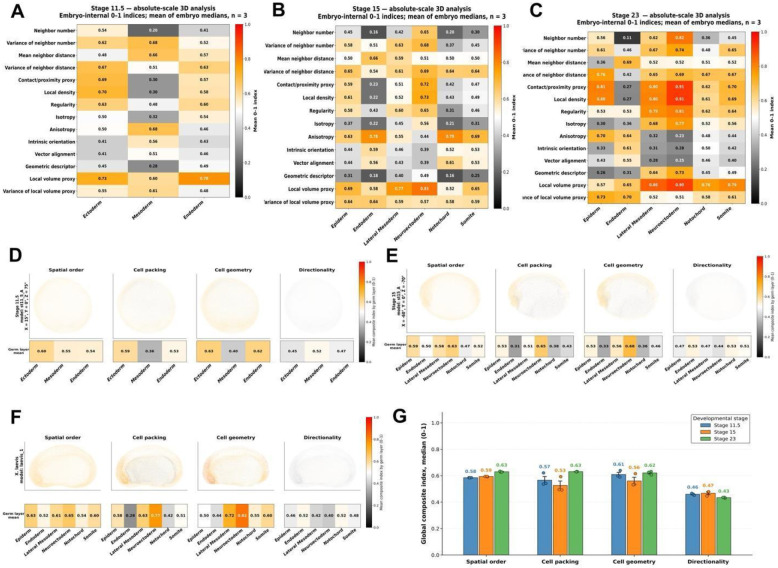
Spatiotemporal topological mapping reveals shifting physical and architectural dynamics during *Xenopus laevis* development. (A-C) Tissue-specific architectural profiles. Heatmaps compiling the median of 14 distinct 3D local neighborhood metrics across embryonic germ layers for (A) gastrulation (St. 11.5), (B) neurulation (St. 15), and (C) larval stage (St. 23) (n = 3 per stage). (D-F) Spatial projection of composite organizational indices. The 14 morphometric features are integrated into four functional macro-categories: Spatial order, Cell packing, Cell geometry, and Directionality. These composite indices are projected as continuous spatial gradients onto 3D embryo coordinates (top) and quantified across tissues (bottom) for (D) St. 11.5, (E) St. 15, and (F) St. 23. (G) Global architectural dynamics. Multi-parametric comparison of the global composite indices across the three developmental stages. This global quantification tracks the macro-scale shifts in physical tissue topology, highlighting the progressive structural reorganization of the embryo over time.

Beyond absolute cell numbers and volumetric tissue allocation, we sought to determine how the spatial organization of cells evolves during development and whether this topological architecture is conserved across species. We first mapped the spatiotemporal progression of tissue architecture during *Xenopus laevis* development by extracting 14 local 3D neighborhood metrics from our segmentation data (Figure 5). By integrating these metrics into composite indices (spatial order, cell packing, cell geometry, and directionality), we tracked macro-scale shifts in physical tissue topology.

During embryonic development, certain tissues maintained highly conserved architectural profiles, such as the epidermis and endoderm. Conversely, other structures exhibited marked reorganization throughout the process, notably the neuroectoderm and somites, which showed significant variations in cell packing and cell geometry. Globally, across the three analyzed stages, cell packing and cell geometry were the two properties that manifested the greatest tendency for morphological change during development, with the most pronounced transition occurring precisely during neurulation (St. 15).

**Figure 6.**
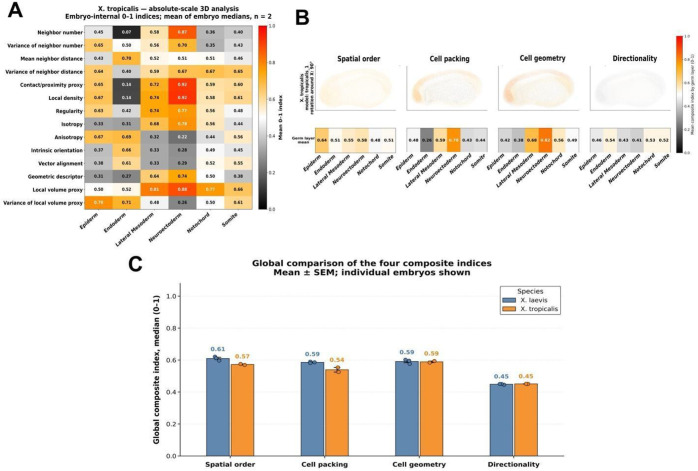
Interspecies architectural fingerprinting reveals conserved topological organization between *X. laevis* and *X. tropicalis*. **(A)** Heatmap of 14 local 3D neighborhood metrics across *X. tropicalis* larval tissues (St. 23, n = 2). **(B)** Integration of these metrics into four composite organizational indices (Spatial order, Cell packing, Cell geometry, Directionality), mapped as 3D spatial gradients and quantified by tissue. **(C)** Interspecies comparison reveals highly conserved macro-scale spatial organization between *X. laevis* and *X. tropicalis*, despite differences in absolute embryo volume and cell counts.

We next investigated whether this spatial organization is evolutionarily conserved by comparing *X. laevis* and *X. tropicalis* at the larval stage (St. 23) (Figure 6). Despite the 1.38-fold difference in overall cellular packing density and the striking variations in total embryonic volume, the local geometric relationships and composite organizational indices were highly similar between the two species and showed no statistically significant differences (Mann-Whitney p ≥ 0.200) between *X. laevis* and *X. tropicalis*. These “architectural fingerprints,” which quantify the topological arrangement of the embryonic germ layers, displayed overlapping median values in both models. This demonstrates a robust conservation of macro-scale spatial organization that is established independently of absolute embryonic scale or cell count. These findings align with the concept of a species-independent archetype of tissue dynamics proposed by Morishita et al. (2023), demonstrating that morphogenetic dynamics across different vertebrate models can be mapped onto one another via scale-invariant topological transformations.

## DISCUSSION

### Scaling of Cell Number Without Disruption of Architecture

Our quantitative 3D mapping of *Xenopus* development from gastrula to early tailbud reveals that embryonic scaling is not a simple proportional multiplication of cell numbers. *X. laevis* is 2.29-fold larger in volume and 1.66-fold higher in cell count than *X. tropicalis*, yet the spatial organization and topological architecture of the embryo are strikingly conserved. The discrepancy between volumetric scaling (2.29×) and cell number scaling (1.66×) is resolved by the higher packing density of *X. tropicalis* cells (1.38×). This pattern is consistent with the known biology of *Xenopus* cell size determination: cell size is primarily set by egg size, while nuclear size scales with genome content (ploidy), leading to different nuclear-to-cytoplasmic ratios in the two species (Levy & Heald, 2010; Miller et al., 2023).

These findings add a quantitative dimension to the longstanding observation that *Xenopus* species of different sizes produce proportionally similar larvae. They demonstrate that the embryo’s cellular “blueprint” is robust to changes in absolute cell number and density—a conclusion that resonates with the broader principle of positional information and tissue scaling, whereby tissue proportions adjust to altered cell numbers through scaling of morphogen gradients and mechanical signals (Čapek & Müller, 2019; Wolpert, 1969; Almuedo-Castillo et al., 2018).

### Conserved Proportionality of Germ Layer Allocation

The conservation of fractional germ layer composition across species is particularly noteworthy. Our data show that the proportional allocation of cells to ectoderm, mesoderm, and endoderm is maintained between *X. laevis* and *X. tropicalis* at equivalent developmental stages, despite a 1.66-fold difference in absolute cell numbers. This proportionality implies that the inductive signals and cell fate decisions governing germ layer specification operate in a scale-invariant manner. This is consistent with the observation that scale-invariant patterning by size-dependent inhibition of Nodal signaling maintains correct tissue proportions in zebrafish embryos even when total cell number is experimentally reduced (Almuedo-Castillo et al., 2018).

The progressive increase in the mesodermal fraction from gastrula to tailbud stages, observed in both species, reflects the sequential recruitment of cells from the deep neurectoderm into mesodermal positions—a process documented in detail by Cooke (1979b) in *X. laevis* and now shown here to occur proportionally in *X. tropicalis* as well.

### Topological Conservation as an Architectural Fingerprint

The conservation of composite morphometric indices between species—our “architectural fingerprinting”—represents the most striking evidence for shared design principles. These indices capture not just the number of cells in each tissue, but their spatial arrangement relative to each other and to the embryo’s body axes. The overlapping values between *X. laevis* and *X. tropicalis* suggest that the embryo is organized according to a conserved spatial template that is independent of absolute size. This concept aligns with the “archetype” of developmental tissue dynamics proposed by Morishita et al. (2023), who showed that morphogenetic dynamics in tissues of different species can be mapped onto each other through mathematical transformations, revealing species-independent organizational principles.

The conservation of local packing geometry—nearest-neighbor distances, angular distributions of cell-cell contacts—further supports the idea that cells in both species follow the same local packing rules. This may reflect shared mechanical constraints on cell arrangement in densely packed embryonic tissues, as well as conserved cell adhesion and cortical tension properties (Heisenberg & Bellaïche, 2013).

### Limitations and Future Directions

Our morphometric indices, while capturing large-scale topological features, do not resolve finer-scale spatial patterns such as cell polarity, intercalation behaviors, or tissue-level anisotropy. Integration of single-cell and spatial transcriptomic data will be essential for linking quantitative cellular metrics to molecular identity and fate decisions. Together, our results establish a quantitative framework for understanding how *Xenopus* embryos of different sizes achieve the same body plan. The conservation of cellular architecture—proportional tissue allocation, spatial order, packing geometry, and fractional mitotic activity—across species with substantially different absolute cell numbers reveals that early vertebrate embryos follow shared quantitative design principles. These principles ensure that cell number scales without disrupting the fundamental organization of the developing body plan.

## MATERIALS AND METHODS

### Embryo Preparation and Staging

All animal experiments were conducted following the rules and regulations of the Harvard Medical School IACUC (Institutional Animal Care and Use Committee #1365–6). Females of Xenopus laevis were primed and boosted with 10 U and 700 U of HCG (Human chorionic gonadotrophin, Chorulon, Merck, Germany), respectively. Testes were collected after male euthanasia. Eggs were laid and then collected in 1X MMR; excess media was removed, and fertilized in vitro with a sperm solution in 1X MMR. Fertilized eggs were de-jellied using 2% W/V cysteine at pH 7.8 in 0.1X MMR. Embryos were cultured in 0.1X MMR at 18–22 °C and staged according to the Niewkoop and Faber (1994) table. X. tropicalis embryos were obtained by in vitro fertilization according to Lane et al. (2022). Embryos from both species at stages 11.5, 15, and 23 were fixed in MEMFA (0.1 M MOPS (pH 7.4), 2.0 mM EGTA, 1.0 mM MgSO_4_) with 4% v/v Formaldehyde overnight at 4 °C.

### Pigment Removal and Nuclear Staining

Fixed embryos were bleached with PBS containing 5% hydrogen peroxide and 0.1% Tween-20 (v/v) under fluorescent light to remove melanin pigmentation. Nuclei were stained with SYTOX Green (1:10,000), DAPI (5 μg/mL), or TO-PRO3 (1 μM) in PBS with 0.02% Triton X-100. Stained embryos were extensively washed with PBS containing 0.02% Triton X-100 (v/v).

### iDISCO Tissue Clearing

After washing, the embryos were dehydrated in H2O/ethanol (v/v; 30%, 50%, 70%, 90%, and 100%) for 24h. After dehydration, embryos were transferred to ethyl cinnamate (ECi, 100%).

### Imaging

Cleared embryos were imaged using a Nikon Ti2 inverted microscope equipped with a Yokogawa CSU-W1 spinning disk confocal system and a Hamamatsu ORCA-Fusion sCMOS camera. Whole-embryo overviews were acquired using a Plan Apo λ 10×/0.45 NA objective, while regional details were captured with a 20×/0.75 NA objective. Volumetric 3D imaging (Z-stacks) was performed with a 2 μm step size (e.g., 480 optical slices for the 10× overview). Fluorescence excitation was achieved using a 640 nm laser, and emission was collected through a Chroma ET705/72m bandpass filter.

### Image Analysis and Segmentation

3D nuclear segmentation was performed using StarDist3D (Weigert et al., 2020). Prior to inference, images were resampled to isotropic voxels (2.0 μm × 2.0 μm × 2.0 μm) and enhanced via a custom percentile-based normalization and gamma correction. To optimize memory, inference was run on overlapping image tiles (15%) using conservative thresholds (probability = 0.30, NMS = 0.20). Finally, 3D spatial coordinates and volumetric data were extracted via scikit-image to guide the germ layer annotation based on brightfield boundaries.

### Layer annotation

To identify the embryonic germ layers, manual annotations were performed using the multi-dimensional image viewer Napari. Anatomical and histological references were based on The Early Development of Xenopus laevis: An Atlas of the Histology (Hausen & Riebsell, 1991). To optimize the annotation workflow and increase efficiency without compromising segmentation quality, manual annotation was performed on every fourth optical slice, resulting in an 8 μm Z-spacing between annotated planes. This sampling strategy significantly reduced the total number of manually processed frames while maintaining a high-fidelity representation of the tissue architecture. Finally, each annotated layer was exported and saved individually as a separate file for downstream processing.

### Topological mapping

The three-dimensional spatial organization of embryonic tissues was quantified from extracted nuclear coordinates (x, y, z), after anisotropic rescaling to correct for differences between axial and lateral voxel dimensions and to represent the true physical space. For each nucleus, local neighborhoods were defined within a fixed Euclidean radius, and nuclei with fewer than a minimum number of neighbors were excluded to reduce edge effects and numerical instability. From the relative vectors connecting each nucleus to its neighbors, local density was estimated as the number of neighboring nuclei, while spatial proximity, used as a proxy for cellular contact, was calculated from the inverse squared inter-nuclear distances and log-transformed to stabilize variance. The regularity of local cell organization was quantified by combining radial spacing uniformity, based on the coefficient of variation of neighbor distances, with angular uniformity derived from the distribution of relative unit vectors. Local microenvironment geometry was further characterized by principal component analysis of the 3D covariance matrix of neighbor vectors, from which isotropy was defined as the ratio of the smallest to the largest eigenvalues, and directional anisotropy was calculated from the two largest eigenvalues to estimate tissue alignment. To enable comparison across samples and integration into spatial maps and heatmaps, all continuous metrics were clipped at the 5th and 95th percentiles and normalized to a dimensionless 0–1 scale using Min–Max rescaling.
